# Psychometric properties testing of a Cantonese version of the Life-Space Assessment in people with stroke

**DOI:** 10.1038/s41598-021-00140-w

**Published:** 2021-10-18

**Authors:** Lily Y. W. Ho, Claudia K. Y. Lai, Shamay S. M. Ng

**Affiliations:** 1 School of Nursing, The Hong Kong Polytechnic University, Hung Hom, Kowloon, Hong Kong SAR China; 2 Department of Rehabilitation Sciences, The Hong Kong Polytechnic University, Hung Hom, Kowloon, Hong Kong SAR China

**Keywords:** Stroke, Health care

## Abstract

The Life-Space Assessment (LSA) advances measurements of mobility by determining the extent of the spatial area in which a person moves in real life. Yet there is no Cantonese version of the LSA. This study aimed to translate and culturally adapt the LSA into Cantonese (C-LSA) and examine its psychometric properties in people with stroke. Psychometric properties were examined in 112 people with stroke. The life-space of stroke survivors was compared with that of healthy older people with and without depressive symptoms. The content validity of the C-LSA was good. The Cronbach’s α was 0.73. The test–retest reliability was 0.95. The standard error of measurement was 4.21 and the minimal detectable change was 11.66, without any ceiling or floor effects in the C-LSA composite score. The composite score correlated significantly with the Fugl-Meyer Assessment of lower extremities score (r_s_ = 0.31), the Five Times Sit-To-Stand time (r_s_ =  − 0.43), and the Frenchay Activities Index score (r_s_ = 0.48). People with stroke had significantly lower C-LSA composite scores than healthy older people. Depressive symptoms worsened the composite and assisted life-space scores only of people with stroke. The C-LSA is a reliable and valid tool for measuring life-space in stroke populations.

## Introduction

With an aging population, the number of strokes is expected to increase. Motor impairments^1^ and limited functional mobility^2^ commonly appear after a stroke. Limitations of functional mobility negatively affect a person’s physical, social, and psychological well-being^3^. One of the important goals of stroke rehabilitation is to enhance the ability of people with stroke to move around in daily life. Such capabilities are necessary for participating in activities and reintegrating in the community during the recovery process. Although there are many tools for measuring the walking capacity of people with neurological diseases, such as the Timed Walk Test and the Rivermead Mobility Index^4^ in clinical settings, most of them focus on assessing walking ability in the laboratory or clinic but overlook how people with stroke deal with their actual environments despite their disabilities. Holistic care for people with stroke should not only emphasize physical ability but also concern the person as a whole and consider the interactions between the person and the environment.

Life-space refers to the size of the spatial area in which a person moves purposefully within his/her physical and geographical capacity. The concept addresses the interaction between people and their environments^5^. Thus, assessments of mobility should be transferable from the laboratory or clinic to real life situations in clinical settings. Understanding the actual mobility of people with stroke in real life facilitates goal setting in stroke rehabilitation.

Several tools are available to assess the life-space of older people. A life-space diary, for example, requires the respondent to record life-space-related observations in diary format^6^. That can be challenging for people with motor impairment. The Life Space Questionnaire focuses on the life-space occupied over the previous 3 days^7^. That may not reflect the real life situation. The Life-Space Assessment (LSA) measures a person’s extent and frequency of movement during the previous 4 weeks, and also takes into consideration the assistance required by that person^8^. It is suitable for use in stroke populations as it is concerned with both functioning and disability, as well as with contextual factors as described in the International Classification of Functioning, Disability, and Health framework proposed by the World Health Organization^9^.

The LSA, which is a self-reported scale consisting of five levels, was developed by Baker et al.^8^ to quantify mobility. It addresses the full continuum of mobility and changes in mobility based on the relationship between a person and the environment. Its test–retest reliability is excellent, with intra-class correlation coefficients (ICCs) of 0.95–0.97 over 2 weeks for the original LSA in community-dwelling older people^8^. LSA scores correlate positively with physical performance and self-reported health (r_s_ = 0.42–0.60), and negatively with functional measures, comorbid conditions, and depressive symptoms (r_s_ =  − 0.91 to − 0.41) in older people^8^. Among outpatients with stroke in a medical center, the test–retest reliability in terms of the kappa statistic has been shown to be 0.99, and LSA scores also correlate significantly with the scores of Functional Ambulation Categories (r = 0.85) and the mobility subscale of the Functional Independence Measure (r = 0.76)^10^. A lower life-space score has been found to be significantly associated with stroke in older Mexican-Americans^11^.

Life-space reveals the physical decline of a person in the environment, but the use of life-space is limited in stroke populations. Depressive symptoms could affect the extent of the life-space of people with stroke^10^. The English version of the LSA^8^ has been translated into several languages, including French-Canadian^12^, Japanese^13^, Spanish^14^, Portuguese^14^, Finnish^15^, Swedish^16^, Brazilian-Portuguese^17^, Korean^10^, and Chinese^18,19^. However, different spoken languages and conceptual inequivalence make some words in the English and Chinese versions inapplicable to the local population in this study.

This study aimed to (1) translate and culturally adapt the LSA into Cantonese; (2) test its psychometric properties, including content validity, internal consistency, test–retest reliability, standard error of measurement, minimal detectable change, ceiling and floor effects, and correlations with motor performance and the performance of daily activities of Chinese people with stroke; and (3) examine the differences in LSA scores between people with stroke and healthy older people, with and without depressive symptoms in the Hong Kong Special Administrative Region.

## Methods

### Phase 1: Translation and cultural adaptation

The original version of the LSA^8^ was translated and back-translated according to the guidelines recommended by Beaton et al.^20^ A bilingual nurse familiar with the concepts in the LSA and a professional translator unfamiliar with the concepts first independently translated the LSA from English to Cantonese. The LSA was culturally adapted by using “district” in place of “town” in the original. The translators’ two versions were synthesized into one “common” translation, which was then back-translated from Cantonese into English independently by two bilingual speakers with no medical background and who were unfamiliar with the concepts.

The back-translated versions were compared to the original English version to check whether there was any loss in meaning. The semantic, experiential, conceptual, and idiomatic equivalence of the translated LSA were evaluated by a panel of five professionals, including nurses, physiotherapists, and a translator. A 4-point Likert scale with 1, 2, 3, and 4 representing “not relevant”, “somewhat relevant”, “quite relevant”, and “highly relevant”, respectively, was used to assess each item in the translated version. Items with a rating of 3 or 4 were considered relevant, while those rated 1 or 2 were regarded as irrelevant. The use of a particular word did not well reflect the meaning of “place” in life-space 3 (“places in your neighborhood, other than your own yard or apartment building”). That word was revised by agreement among the expert committee. A pilot version was thus formed and was tested on 40 Chinese people with stroke^20^.

The first two participants thought that the pilot version was not clear, and they suggested to include examples to explain some of the terms used. Several footnotes were therefore added as explanations (Table 1). After these revisions, the revised LSA was administered to the remaining 38 participants. Among them, 37 (97.4%) agreed that the revised LSA was comprehensible and clear. This again revised version (C-LSA) was considered ready for psychometric testing.

**Table 1:** Footnotes added to explain the terms in the Life-Space Assessment.

Terms in the Life-Space Assessment	Footnotes added
The room where you sleep	A bed for sleeping is used in case the respondent has no room in which to sleep
Other rooms of your home	For example, the bathroom
Neighborhood	For example, a nearby supermarket or market
Within your district	For example, the Hong Kong Special Administrative Region is divided into 18 districts. If a respondent lives in Tsuen Wan District, “within your district” refers to places within Tsuen Wan District
Outside your district	For example, if a respondent lives in Tsuen Wan District, “outside your district” refers to places outside Tsuen Wan District

### Phase 2: Testing of psychometric properties

#### Setting and sampling

A convenience sample of people with stroke and healthy older people was recruited from local self-help groups and a non-governmental organization offering community services in the Hong Kong Special Administrative Region in 2019. The inclusion criteria for people with stroke were: (1) aged ≥ 55; (2) a Cantonese speaker; (3) ambulatory; (4) community-dwelling; and (5) with stroke diagnosed for 1 year or more. The exclusion criteria included: (1) having any other neurological disease; (2) an unstable health condition; or (3) having a history of transient ischemic attacks. The inclusion criteria for healthy older people included (1) aged ≥ 55; (2) a Cantonese speaker; (3) ambulatory; and (4) community-dwelling. The exclusion criteria were the same as for people with stroke.

All eligible recruits were invited to take part in a face-to-face interview once in the university or the participating non-governmental organization. It has been recommended to have 2–20 participants per item with a minimum of 100 participants to validate a scale^21^. With five levels of life-space, it was estimated that at least 100 participants would be needed. There were no previous studies quantifying the test–retest reliability of the LSA in people with stroke. The lowest published ICC in validation studies of the LSA in other languages with other populations was 0.70^12,14,15,17,19,22^. To establish the test–retest reliability of the instrument, 27 people with stroke were needed with two observations per participant under the alternative hypothesis at a power of 0.80 and a significance level of 0.05. Therefore, the first 27 people with stroke were invited to take part in a reassessment at a mean of 7.22 ± 0.51 days later. All of the assessments were conducted by the same assessor.

To compare the differences between people with stroke and healthy older people at an effect size of 0.50, a power of 0.80, and a significance level of 0.05, a sample size of 64 people with stroke and 64 healthy older people would be needed using a t test in GPower 3.1.

#### Measurement

Life-space was assessed using the C-LSA. It assessed mobility habits from within the home environment (Level 1) to beyond the district (Level 5) over the previous 4 weeks. It first assessed whether the respondent had attained each of the five levels with a yes/no response. The frequency with which the 5 levels was reached was assessed with four response choices: < 1 time/week, 1–3 times/week, 4–6 times/week, or daily. The requirement for assistive devices and/or human assistance was also measured with three response choices: yes, no, or don’t know. A composite score ranging from 0 to 120 was then calculated from the responses on life-space level, frequency, and the requirement for assistive devices or human assistance. Higher scores indicated a wider life-space. The cut-off score was 60^23^. The maximal life-space, independent life-space, and assisted life-space were also assessed. The maximal life-space reflected the greatest distance achieved with the use of any assistive device and/or human assistance when necessary. The independent life-space assessed the highest level of life-space achieved without using any assistive device or human assistance. The assisted life-space assessed the highest level of life-space achieved with the help of assistive devices but without human assistance. The maximal, independent, and assisted life-space scores ranged from 0 to 5. They were generated with the Statistical Package for the Social Sciences Syntax provided by the authors of the original LSA^8^. The LSA is known to be reliable in a stroke population^10^.

#### Instruments used for validation

Since life-space was found to correlate with physical performance, functional measures, and depressive symptoms in older people^8^, this study validated the C-LSA with Fugl-Meyer Assessment—lower extremity, Five Times Sit-To-Stand Test, and Frenchay Activities Index, and compared the life-space between those with and without depressive symptoms using the Geriatric Depression Scale.

Fugl-Meyer Assessment for the lower extremities was used to assess motor function including movements, reflex actions, and coordination^24^. It consists of 17 items scored 0, 1, or 2, indicating “cannot perform”, “partially performs”, or “fully performs”, respectively. Total scores range from 0 to 34. Higher scores represent better motor performance. The test–retest reliability over 5–10 days has been shown to be 0.94^25^.

The Five Times Sit-To-Stand Test was used to assess functional muscle strength. It measures the time needed to complete five sit-to-stand maneuvers in succession. It has excellent test–retest reliability (ICC = 0.99–1.00) and intra-rater reliability (ICC = 0.97–0.98)^26^. The test is significantly associated with the muscle strength of the knee flexors (affected or unaffected), with Spearman ρs ranging from − 0.75 to − 0.83 among people with stroke^26^.

Performance of daily activities was assessed using the Chinese version of the Frenchay Activities Index^27^, as suggested by the American Heart Association’s Classification of Stroke Outcome Task Force^28^. The 15-item scale assesses the frequency of participation in chores and activities. Total scores range from 0 to 45, with higher scores indicating more frequent participation^27^. This tool has shown good test–retest reproducibility (ICC = 0.89)^29^ and it is significantly correlated with the modified Nottingham Extended Activities of Daily Living Scale (*⍴* = 0.80) in people with stroke^30^.

Depressive symptoms were measured using the Chinese version of the 15-item Geriatric Depression Scale^31,32^, as suggested by the American Heart Association’s Classification of Stroke Outcome Task Force^28^. The Geriatric Depression Scale consists of 15 yes/no questions about the feelings of the respondents over the past week. Total scores range from 0 (no depressive symptoms) to 15 (severe depressive symptoms) with a cut-off point of 8^33^. In people with stroke, its internal consistency was 0.78^34^. In people with Parkinson’s disease, internal consistency was 0.90 and test–retest reliability was 0.94^35^. In depressed people, Cronbach’s α was 0.84 and test–retest reliability was 0.91^36^. Healthy older people were interviewed using only the C-LSA and the Geriatric Depression Scale.

### Ethical considerations

The Declaration of Helsinki was followed, and ethical approval to conduct this study was granted by the Human Subjects Ethics Sub-committee of The Hong Kong Polytechnic University. The objectives, data collection procedure, and issues regarding confidentiality and anonymity were explained to the participants. All of them signed a declaration of written informed consent.

### Data analysis

Version 25 of the Statistical Package for the Social Sciences was used to analyze the data. The characteristics of the participants and their life-spaces were summarized in descriptive statistics. The differences in demographic data between people with stroke and healthy older people were compared using an independent sample t-test or a Chi-square test.

To measure the reliability and responsiveness of the C-LSA, the test–retest reliability of each level of life-space, composite score, maximal life-space, independent life-space, and assisted life-space was measured by computing an ICC_3,1_, where < 0.50 indicated poor, 0.50–0.75 was taken as moderate, 0.75–0.90 indicated good, and > 0.90 indicated excellent reliability^37^. Model 3 was chosen to fix the rater effect. The standard error of measurement was calculated as S × $\sqrt {1 - r}$^38^, while minimal detectable change at a 95% confidence interval was calculated as S × $\sqrt {2\left( {1 - r} \right)}$ × 1.96^39^, where S was the standard deviation of the composite score at baseline while r was the test–retest reliability coefficient. Cronbach’s α coefficient was used to assess the internal consistency of the instrument. A Cronbach's α value of > 0.70 was considered acceptable^40^. Ceiling or floor effects were regarded as present when > 15% of the participants got the highest or lowest score, respectively^41^.

Regarding the validity, both item-level and scale-level content validity indices were calculated. An item-level content validity index score of ≥ 0.78 and an scale-level content validity index score of ≥ 0.90 are regarded as good^42^. Kolmogorov–Smirnov tests showed that not all data were normally distributed. Correlations between the C-LSA and the Fugl-Meyer Assessment for the lower extremities, Five Times Sit-To-Stand Test, and Frenchay Activities Index were examined using Spearman correlation coefficients. A coefficient of 0.10–0.39 was considered weak, 0.40–0.69 was moderate, 0.70–0.89 was strong, and over 0.90 was taken as representing a very strong correlation^43^. A *p* value of less than 0.017 (0.05/3) was regarded as statistically significant after a Bonferroni adjustment was made. Missing data were excluded pairwise.

The life-space scores of people with stroke and healthy older people were compared using Mann–Whitney tests. A *p* value of < 0.05 was considered significant.

## Results

### Participant characteristics

One hundred and twelve people with stroke and 65 healthy older people were recruited. In the end, 66 out of the 112 people with stroke were randomly drawn by the computer for a comparison with 65 healthy older people.

The participants were 64.15 ± 5.79 years old with an average time since stroke of 73.60 ± 57.43 months. A summary of their demographic and life-space characteristics is shown in Table 2.

**Table 2:** Demographic and life-space characteristics of the participants (N = 112).

	Mean (Standard deviation)
Life-space composite score	69.8 (18.45)
Maximal life-space score	5.00 (0.00)
Independent life-space score	2.44 (1.74)
Assisted life-space score	4.36 (1.30)
	**N (%)**
Gender: Male	74 (66.1)
**Marital status**	
Single	7 (6.3)
Married	89 (79.5)
Separated/divorced	8 (7.1)
Widower/widow	8 (7.1)
**Employment status**	
Employed	6 (5.4)
Unemployed/retired	106 (94.6)
**Mobility status**	
Walks unaided	25 (22.3)
Walks with aid	87 (77.7)

### Reliability and responsiveness

The test–retest reliability coefficients are shown in Table 3. The standard error of measurement of the composite score was 4.21 and the minimal detectable change was 11.66. The Cronbach’s α coefficient was 0.73. There were no ceiling or floor effects in the composite score. Only one of the 112 people with stroke (0.9%) got the highest score and none got the lowest composite score. One hundred percent got the maximum maximal life-space score, but for the independent life-space score the figure was 26.8%, and for the assisted life-space score it was 78.6%. Thus, ceiling effects were present in the maximal, independent, and assisted life-spaces. No one got the lowest maximal life-space score, 12.5% got the lowest independent life-space score, and 0.9% (one person) got the lowest assisted life-space score. Therefore, no floor effects were present in the maximal, independent, and assisted life-spaces.

**Table 3:** Intraclass correlation coefficients (ICC_3, 1_) of the Cantonese version of the Life-Space Assessment (n = 27).

	Intraclass correlation coefficient (95% confidence interval)
Life-space level 1 subscore	0.73 (0.48–0.87)
Life-space level 2 subscore	0.67 (0.40–0.84)
Life-space level 3 subscore	0.85 (0.69–0.93)
Life-space level 4 subscore	0.75 (0.53–0.88)
Life-space level 5 subscore	0.81 (0.62–0.91)
Life-space composite score	0.95 (0.90–0.98)
Maximal life-space score	Zero variance
Independent life-space score	0.95 (0.90–0.98)
Assisted life-space score	1.00 (1.00–1.00)

### Validity

The item-level content validity indices of life-spaces 1, 2, 4, and 5 were 1.00. That of life-space 3 was 0.80. The scale-level content validity index of the LSA was 0.96.

Data for the Fugl-Meyer Assessment for the lower extremities were missing for one person with stroke, while for the Five Times Sit-To-Stand Test data were missing for two people with stroke. The C-LSA was correlated with the Fugl-Meyer Assessment for the lower extremities (r_s_ = 0.31, *p* = 0.001), the Five Times Sit-To-Stand Test (r_s_ =  − 0.43; *p* < 0.001), and the Frenchay Activities Index (r_s_ = 0.48; *p* < 0.001).

### Comparison between people with stroke and healthy older people

The 66 people with stroke were aged 64.85 ± 6.60 while the 65 healthy older people were aged 67.02 ± 6.97 (*p* = 0.070). Fewer participants were employed in the group of people with stroke than in the group of healthy older people (4.5% vs. 20.0%, *p* = 0.007), and more participants required walking aids in the former group than in the latter group (77.3% vs. 9.2%, *p* < 0.001).

The comparisons between the life-space scores of people with stroke and those of healthy older people are shown in Table 4. In the group of people with stroke, 21 had depressive symptoms and 45 had no depressive symptoms. In the group of healthy older people, 10 had depressive symptoms and 55 had no depressive symptoms. Only in the group of people with stroke did participants with depressive symptoms have significantly lower composite and assisted life-space scores. The comparisons of the life-space scores of people with stroke and those of healthy older people with and without depressive symptoms are shown in Table 5.

**Table 4:** Comparison of life-space scores between people with stroke and healthy older people.

	Median (interquartile range)		Mann–Whitney U	Z	*p*
	People with stroke (n = 66)	Healthy older people (n = 65)			
Life-space composite score	69.00 (31.00)	100.00 (30.00)	848.50	− 5.982	< 0.001**
Maximal life-space score	5.00 (0.00)	5.00 (0.00)	1980.00	− 2.288	0.022*
Independent life-space score	2.00 (2.50)	5.00 (0.00)	716.00	− 7.237	< 0.001**
Assisted life-space score	5.00 (3.00)	5.00 (0.00)	1649.50	− 3.386	0.001**

*p < 0.05, **p < 0.01.

**Table 5:** Comparison of life-space scores between people with stroke and healthy older people with and without depressive symptoms.

	People with stroke (n = 66)					Healthy older people (n = 65)				
	Median (interquartile range)		Mann–Whitney U	Z	*p*	Median (interquartile range)		Mann–Whitney U	Z	*p*
	With depressive symptoms (n = 21)	No depressive symptoms (n = 45)				With depressive symptoms (n = 10)	No depressive symptoms (n = 55)			
Life-space composite score	54.00 (32.50)	74.00 (26.25)	297.50	− 2.412	0.016*	83.00 (26.88)	100.00 (24.00)	209.00	− 1.212	0.226
Maximal life-space score	5.00 (0.00)	5.00 (0.00)	472.50	0.000	1.000	5.00 (0.00)	5.00 (0.00)	266.00	− 0.354	0.723
Independent life-space score	1.00 (1.00)	2.00 (4.00)	369.00	− 1.471	0.141	5.00 (0.50)	5.00 (0.00)	255.00	− 0.606	0.545
Assisted life-space score	4.00 (3.00)	5.00 (0.00)	307.00	− 2.872	0.004**	5.00 (0.00)	5.00 (0.00)	266.00	− 0.354	0.723

*p < 0.05, **p < 0.01.

## Discussion

This has been the first test of an LSA translated into Cantonese and culturally adapted for use in a stroke population. The C-LSA showed good content validity, internal consistency, and test–retest reliability without ceiling or floor effects for its composite score. The composite score correlated weakly with the Fugl-Meyer Assessment score for the lower extremities, and moderately with the Five Times Sit-To-Stand time and the Frenchay Activities Index scores. People with stroke had a significantly lower composite score than healthy older people. Depressive symptoms had significant effects on the composite and assisted life-space scores only of people with stroke.

The life-space composite scores in this study were comparable to those of a study conducted in Korea (mean 62.20–63.15)^10^. The infrastructure in the geographical location of a study may play a role in the life-space scores obtained in that study. In a cosmopolitan city with high population density, people live in small apartments in high-rise buildings. There is relatively little personal space for movement. Yet highly developed cities normally have easy-to-access public transport and an environment that allows people with physical disabilities to move around in the city. That might explain why the life-space results of people with stroke in this study were similar to those in Yang et al.’s study^10^. The life-space composite scores of people with stroke ranged from 33.70 to 70.55 in the US^44^. The wide range in those scores might be due to the presence of spatial neglect in the participants in that study^44^. In Japan the score was 42.1^45^. In that study, the participants were receiving daycare services; therefore, their mobility in the community might have been reduced. Today, 55% of the global population lives in cities, and by 2050 the United Nations predicts that the proportion will reach 68%^46^. Therefore, it is important to have a tool to measure life-space in cosmopolitan cities.

Content validity has been tested only in the French-Canadian^12^, Brazilian-Portuguese^17^, and Chinese versions of the instrument^18^. The meaning of 86% of the items in the French-Canadian version was found to correspond well with that of the original version ^12^, while professionals agreed entirely with the content of the Brazilian-Portuguese version^17^. The item-level and scale-level content validity indices of this Cantonese version were similar to and greater than those of a Chinese version, respectively (item-level 1.0; scale-level 0.86)^18^. This indicates that the concepts of life-space were well represented by the items in the C-LSA in this study.

Previous validation studies have shown good to excellent ICCs for the composite score of LSA^8,12,16–19^ and this was also the case in this study. The ICCs of the sub-scores, which ranged from moderate to good in this study, were lower than those of the Brazilian-Portuguese version (ICC = 0.92–0.99) although the retest interval was very similar^17^. A possible explanation for this result might be that an important festival, the Chinese New Year, was held during the data collection period of this study, leading to an increase in the frequency of the activities of the participants, so there might have been some real changes in their life-space. In retrospect, collecting data for the test–retest interval over a period spanning the Chinese New Year was a mistake. Regarding the reliability of the maximal, independent, and assisted life-spaces, the ICCs in this study were higher than those observed in the French-Canadian (ICC = 0.76–0.84)^12^, Spanish (ICC = 0.37–0.63)^14^, and original versions (ICC = 0.49–0.81)^8^. Since maximal, independent, and assisted life-spaces take into consideration only an individual’s level of independence in reaching particular life-spaces, these findings may imply that people with stroke in this study experienced fewer changes in their need for assistive devices and/or human assistance than users of powered mobility devices in Auger et al.’s study^12^ and than the older populations in the studies of Curcio et al.^14^ and Baker et al.^8^.

The standard error of measurement (3.51%) of the composite score in this study was very good^47^. It was very similar to that of the Brazilian-Portuguese version (3.43% over 1 week)^17^ and better than that of the Swedish version (7.58% over a 2-week period)^16^. There might be real changes in the life-space of the participants when the test–retest interval is increased.

The minimal detectable change was acceptable as it was within 10% of the total possible range of scores^48^. It implied that the LSA score could reflect a real change that was not due to a measurement error^39^ and that LSA scores could be used to evaluate the effectiveness of therapies such as mobility training in stroke rehabilitation.

Like the French-Canadian^12^, Finnish^15^, and Brazilian-Portuguese versions^17^, the composite score in the C-LSA had no significant ceiling or floor effect. It did, however, show ceiling effects for the maximal, independent, and assisted life-spaces. In that respect, it resembled the Finnish version^15^. In the French-Canadian version a ceiling effect was found for the maximal life-space, and floor effects were found for the independent and assisted life-spaces^12^. The LSA can discriminate at both ends of the composite scale, but the maximal, independent, and assisted life-spaces may not be responsive to differences at the positive end. Data on the maximal, independent, and assisted life-spaces should be used with caution due to their narrower range of valid scores compared with the composite scores.

The LSA scores were correlated with the scores of the Fugl-Meyer Assessment for the lower extremities, Five Times Sit-To-Stand time, and the Frenchay Activities Index scores. For older populations, life-space is affected by step counts^49^, and greater life-space is associated with better lower extremity function, and upper and lower extremity muscle strength^11^. After a stroke, lower extremity strength is correlated with functional mobility^50^. Lower extremity function and muscle weakness affect mobility and thus life-space. Therefore, consideration can be given to integrating components targeting motor function as well as muscle strength in mobility training. The correlation between life-space and the performance of daily activities was supported by a recent study conducted in Finland, where older adults usually go outdoors to engage in activities, which include shopping, walking as exercise, social visits, and doing errands^51^. There are various reasons for going outdoors, and engaging in activities is one of them. Since going to a place for multiple purposes and the social component of activities facilitate participation^52^, when designing activities consideration could be given to the question of how to attract people with stroke, in order to widen their life-space. Since the aim of stroke rehabilitation is to help people regain the capabilities that they need to resume their normal life, future studies could explore the mediating factors in widening the life-space of people with motor impairment and muscle weakness after a stroke.

Although both people with stroke and healthy older people were not classified as having a restricted life-space, the people with stroke had a significantly lower composite score than the healthy older people. Since the LSA takes into consideration the assistance required by individuals, the larger proportion of people with stroke needing walking aids greatly decreased the composite and independent life-space scores. Although the median assisted life-space score was the same in both groups, the people with stroke had a wider interquartile range of assisted life-space scores and this reflected the fact that they needed assistive devices when moving in the actual environment.

There were no significant differences in maximal life-space and independent life-space scores between the people with stroke and the healthy older people with and without depressive symptoms. This might be because when people needed to reach certain places to fulfill their needs, such as attending follow-up appointments, depressive symptoms might not have had an impact on their ability to do so. In addition, depressive symptoms might not affect an individual’s physical ability to mobilize. The composite and assisted life-space scores were significantly lower for people with stroke with depressive symptoms than for those without such symptoms, but these differences were not found in the healthy older people. According to the International Classification of Functioning, Disability, and Health framework^9^, life-space is possibly mediated by contextual factors. People with stroke perceived that the use of assistive devices such as walking aids could result in social stigma^53^, which might lead to depressive symptoms. They therefore may have less motivation to go far, especially when it is not necessary to go to some places.

In interpreting and applying these results it is important to note that the participants of this study were self-selected. Those who volunteered might have had better mobility or better support to travel to attend the interview for this study. Also, the people with stroke were recruited from local self-help groups. They might already have been relatively active, participating in activities organized by the groups and thus having a greater life-space. These circumstances might limit the generalizability of this study. In addition, as has been explained, the increased activity surrounding the Chinese New Year might have led to unusual changes in life-space. Thus the data on test–retest reliability, standard error of measurement, and minimal detectable change should be interpreted with caution. The size of the sample for comparing the life-space scores between people with stroke and healthy older people with and without depressive symptoms was small; a larger sample size should be adopted in future studies.

## Conclusion

The C-LSA is a reliable and valid tool for measuring life-space in community-dwelling people with stroke in both clinical and research settings. It can be used to evaluate the impact of interventions on their actual daily life. A difference of 11.66 in the composite score indicates an actual change in life-space. People with stroke had significantly lower life-space composite scores than healthy older people, and depressive symptoms lowered the composite and assisted life-space scores of people with stroke.

## Data Availability

All data generated or analysed during this study are included in this published article.
